# Antiepileptogenic and Neuroprotective Effects of *Pergularia daemia* on Pilocarpine Model of Epilepsy

**DOI:** 10.3389/fphar.2017.00440

**Published:** 2017-06-30

**Authors:** Antoine K. Kandeda, Germain S. Taiwe, Fleur C. O. Moto, Gwladys T. Ngoupaye, Gisele C. N. Nkantchoua, Jacqueline S. K. Njapdounke, Jean P. O. Omam, Simon Pale, Nadege Kouemou, Elisabeth Ngo Bum

**Affiliations:** ^1^Department of Animal Biology and Physiology, Faculty of Science, University of Yaoundé IYaoundé, Cameroon; ^2^Department of Biological Sciences, Faculty of Science, University of NgaoundereNgaoundere, Cameroon; ^3^Department of Zoology and Animal Physiology, Faculty of Science, University of BueaBuea, Cameroon; ^4^Department of Biological Sciences, Higher Teachers’ Training College, University of Yaounde IYaounde, Cameroon; ^5^Department of Animal Biology, Faculty of Science, University of DschangDschang, Cameroon; ^6^Institute of Mining and Petroleum Industries, University of MarouaKaele, Cameroon

**Keywords:** antiepileptogenic, antioxidant, neuroprotective, *Pergularia daemia*, pilocarpine, status epilepticus

## Abstract

In this study, we investigated antiepileptogenic and neuroprotective effects of the aqueous extract of *Pergularia daemia* roots (PDR) using *in vivo* and *in vitro* experimental models. In *in vivo* studies, status epilepticus caused by pilocarpine injection triggers epileptogenesis which evolves during about 1–2 weeks. After 2 h of status epilepticus, mice were treated during the epileptogenesis period for 7 days with sodium valproate and vitamin C (standards which demonstrated to alter epileptogenesis), or *Pergularia daemia*. The animals were then, 1 week after status epilepticus, challenged with acute pentylenetetrazole (PTZ) administration to test behaviorally the susceptibility to a convulsant agent of animals treated or not with the plan extract. Memory was assessed after PTZ administration in the elevated plus maze and T-maze paradigms at 24 and 48 h. Antioxidant and acetylcholinesterase activities were determined in the hippocampus after sacrifice, *in vitro* studies were conducted using embryonic rat primary cortical cultures exposed to L-glutamate. Cell survival rate was measured and apoptotic and necrotic cell death determined. The results showed that chronic oral administration of PDR significantly and dose-dependently increased the latency to myoclonic jerks, clonic seizures and generalized tonic–clonic seizures, and the seizure score. In addition, PDR at all doses (from 4.9 to 49 mg/kg) significantly decreased the initial and retention transfer latencies in the elevated plus maze. Interestingly PDR at the same doses significantly increased the time spent and the number of entries in T-maze novel arm. PDR significantly increased the activities of acetylcholinesterase and antioxidant enzymes superoxide dismutase, catalase, and total glutathione and proteins, and decreased malondialdehyde level. Furthermore, PDR increased viability rate of primary cortical neurons after L-glutamate-induced excitotoxicity, in a dose dependent manner. Altogether these results suggest that PDR has antiepileptogenic and neuroprotective effects, which could be mediated by antioxidant and antiapoptotic activities.

## Introduction

Temporal lobe epilepsy (TLE) is a neurological disease that may originate from early precipitating events such as febrile seizures, head trauma, status epilepticus (SE), and infections (Loscher and Brandt, 2010; Kan et al., 2012). The activation of glutamate ionotropic receptors triggers neuronal injury or death predominantly mediated by excessive influx of calcium into neurons through ion channels (Emerit et al., 2004). Therefore, the latter events would be followed by a progressive latent phase of epileptogenesis, leading eventually to spontaneous recurrent seizures and which could also cause cognitive impairment (Marcangelo and Ovsiew, 2007). It was proposed that during epileptogenesis reactive oxygen species overproduction may cause an overwhelming intrinsic antioxidant scavenging capacity, resulting in the development of oxidative stress (Azam et al., 2010), as well as associated tissue injury and apoptotic processes (Todorova et al., 2004; Noor et al., 2015). Despite the high number of antiepileptic drugs currently available, pharmacological agents able to prevent epileptogenesis are lacking. In addition, a high percentage of TLE patients (40%) do not respond to conventional antiepileptic drugs (Kwan and Brodie, 2003; Loscher and Brandt, 2010). Thus, new antiepileptic drugs, possibly with antiepileptogenic properties, are needed. Medicinal plants represent a potential source of such compounds.

According to World Health Organization, about three-quarters of the world population rely upon traditional remedies, mainly medicinal plants (Gilani and Rahman, 2005; Rahmati et al., 2013). Among these, the African and Asian tropical/subtropical plant *Pergularia daemia* (Forsk.) Chiov. (Asclepiadaceae) (*P. daemia*) is used in African and Indian traditional medicine to treat leprosy, poisoning, asthma, anemia, seizures, and mental disorders (Mittal et al., 1962; Karthishwaran and Mirunalini, 2010; Sravani et al., 2012; Sridevi et al., 2014). In Northern Cameroon and in Benin, traditional healers use decoctions of *P. daemia* roots to treat malaria, febrile seizures, epilepsy, mental, and inflammatory disorders (Arbonnier, 2002).

Phytochemically, alkaloids, flavonoids, saponins, triterpenes, tannins and steroidal compounds have been searched in *P. daemia* roots (Bhaskar and Balakrishnan, 2009; Sridevi et al., 2014). Phytochemicals like glucosides and cardenolides in seed, coroglaucigenin, corotoxigenin, uscharidin, and uzarigenin in stem have been identified (Bhaskar and Balakrishnan, 2009; Sridevi et al., 2014). Roots of *P. daemia* were reported to contain β-sitosterol, lupeol, lupeol acetate, and β-amyrin and its acetate (Bhaskar and Balakrishnan, 2009; Sridevi et al., 2014). Organic esters, fatty acids, and phenolic compounds were identified by analysis of the ethanolic extract of the plant (Bhaskar and Balakrishnan, 2009; Sridevi et al., 2014). Various pharmacological properties, including hepatoprotective, antidiabetic, anti-inflammatory, antioxidant, antipyretic, analgesic, and sedative activities have been reported in whole plant extracts (Wahi et al., 2002; Suresh and Mishra, 2008). Aqueous, ethanolic and petroleum ether extracts of *P. daemia* leaves exhibited significant analgesic, antioxidant, antipyretic activities, and antibacterial properties (Suresh and Mishra, 2008). Moreover, active compounds like kaempferol extracted from the roots demonstrated antiepileptic activities (Lokesh, 2009; Sravani et al., 2012).

In the present study, to assess antiepileptogenic effect of *P. daemia* extract, the pilocarpine-induced SE model was used. In this paradigm, SE was induced in mice by intraperitoneal pilocarpine injection. Animals that developed SE for 2 h were selected and received *P. daemia* extract for 7 days (i.e., during the epileptogenesis period). The effects of *P. daemia* were compared to those of sodium valproate (a widely used antiepileptic drug) and vitamin C (an exogenous antioxidant known to inhibit oxidative stress in the brain). These drugs are known to modify epileptogenesis process (Brandt et al., 2003; Xavier et al., 2007; Loscher and Brandt, 2010). During the epileptogenesis period, animals were challenged with pentylenetetrazole (PTZ) in order to assess the susceptibility of animals to seizures and behavioral alterations (Ilhan et al., 2005; Blanco et al., 2009). Effects of the extract on excitotoxicity induced by L-glutamate were assessed on primary cortical neurons in culture. To date, no published study assessed antiepileptogenic and neuroprotective properties of *P. daemia* extract. Therefore, the aim of this study was to assess the putative antiepileptogenic and neuroprotective effects of the aqueous extract of *P. daemia* roots prepared mimicking the traditional healer decoction.

## Materials and Methods

### Drugs and Chemicals

#### *In Vivo* Studies

Vitamin C, PTZ, scopolamine methyl nitrate, diethyl ether, pilocarpine hydrochloride, sodium valproate, Biuret reagent, acetylcholine iodide, 5′5-dithiobis-(2-nitrobenzoic acid) (DNTB), adrenaline, acetic acid, dichromate, hydrogen peroxide (H_2_O_2_), Tris-Hcl, trichloroacetic acid, thiobarbituric acid, sodium phosphate buffer, Griess reagent were purchased from Sigma Chemical Co., St. Louis (United States), while diazepam was purchased from Roche, Neuilly sur-Seine, France. The minimal dose of chemoconvulsant at which 99% of the animals showed a convulsion was determined based on the doses used by other researchers and by a dose-percentage effect curve (Miller and Tainter, 1944; Ahmadiani et al., 2003). Vitamin C and sodium valproate were dissolved in distilled water. All solutions were prepared freshly in the day of the experiment and were administered intraperitoneally at a volume of 10 ml/kg, except for distilled water and aqueous extract of *P. daemia* administered *per os* at the same volume.

#### *In Vitro* Studies

β-D-arabinofuranoside hydrochloride, Hoechst 33342, propidium iodide, 3-(4,5-dimethylthiazol-2-yl)-2,5-diphenyltetrazolium bromide (MTT), oxamate, dimethyl sulfoxide (DMSO), sodium bicarbonate, phosphate buffer saline (PBS), nicotinamide dinucleotide adenine (NAD), diaphorase, L-glutamic acid monosodium salt hydrate, poly-L-lysine, Dulbecco’s modified Eagle’s medium (DMEM), β-mercaptoethanol, lactate, L-glutamine were purchased from Sigma–Aldrich (St. Louis, MO, United States). Fetal bovine serum (FBS) and bovine serum albumin (BSA) were purchased from Gibo/Invitrogen (Carlsbad, CA, United States). Penicillin and streptomycin were purchased from Sanofi-Aventis (Guildford, United Kingdom).

### Plant

#### Collection and Identification

Fresh roots of *P. daemia* were harvested during the month of June 2012 in Mayo-Tsanaga division (Far-North Region, Cameroon). A voucher specimen has been deposited at the Yaoundé national herbarium on the number 7797/SRF/Cam.

#### Preparation of the Aqueous Extract of *P. daemia*

The extract was prepared the day of the experiment, mimicking strictly the procedures used by the traditional healers. The roots were peeled-off, cut to pieces, and air dried at room temperature. Then, dried root samples were grounded into coarse powder. The powder was added to distilled water (5 g in 75 ml) and boiled for 20 min. Following cooling at room temperature, the solution obtained was filtered with Whatman N^o^ 1 filter paper. The filtrate was considered as the stock solution. The amount of dry matter in the extract was determined by evaporating water in a drying oven (50°C). A solid residue (0.37 g) was obtained. The yield of extraction was 7.34%, and the stock solution dose was 49 mg/kg. The other doses used in the study (24.5, 12.3, and 4.9 mg/kg) were obtained by dissolving the stock solution in distilled water at ratios of 1/2, 1/4, and 1/10, respectively.

### Animals

Ninety male or female Swiss albino mice weighting 18–29 g (37–48 days old) were used. They were obtained from Cameroon National Veterinary Laboratory (Lanavet, Garoua, Cameroon) and were housed and bred in the animal facility of the University of Ngaoundere (Ngaoundere, Cameroon). They were kept in a controlled environment, with *ad libitum* access to food and tap water. Animals were maintained on a 12 h/12 h light/dark cycle (lights on at 7:00 a.m.). Animals were acclimated to laboratory conditions before starting the experiments. All procedures were performed in conformance with the Cameroon National Ethical Committee directives (Ref No. FW-IRB00001954, October 22, 1987 under an authorization number CEI-UDo/907/01/2017/T). The study was also performed conforming to international regulations minimizing the number of animals used and avoiding their suffering.

### *In Vivo* Studies

#### Experimental Design

Mice were randomly divided into eight groups of seven animals each. One control group received only distilled water (DW + DW group) and in the other seven groups SE was induced by a single injection of pilocarpine hydrochloride (360 mg/kg, i.p., Sigma–Aldrich). Two hours after SE induction, the following groups were formed: (i) disease group receiving distilled water (10 ml/kg; DW + PILO group); to control the eventual effect of distilled water; (ii) two groups administered either with sodium valproate (300 mg/kg, Sigma–Aldrich) or vitamin C (250 mg/kg, Sigma–Aldrich); (iii) four test groups receiving the doses of *P. daemia* extract (4.9, 12.3, 24.5, and 49 mg/kg) orally, through an intragastric feeding tube. Treatments were administered daily for 7 days. Twenty-four hours after the last administration of the treatments, mice were challenged with PTZ (Blanco et al., 2009). Then, memory was assessed using the following behavioral paradigms: elevated plus-maze (48-h after treatment) and T-maze (72-h after) (Mehla et al., 2010; Taiwe et al., 2015). Animal behavior was recorded by two blinded experimenters. Afterward, mice were sacrificed by decapitation under deep anesthesia with diethyl ether (8%, v/v, Sigma–Aldrich). The brain was dissected out and processed for the quantification of markers of oxidative stress and cholinergic status determination.

#### SE Induction, Behavioral Observations, and Tests

##### SE induction and seizure evaluation

Animals were subjected to epileptogenesis induction by a single intraperitoneal injection of pilocarpine (Turski et al., 1983). The minimal dose of chemoconvulsant at which 99 % of the animals showed seizures was determined based on the available reports (Miller and Tainter, 1944; Turski et al., 1983). This was verified by dose-percentage effect curves obtained in our laboratory; the survival rate was 90%. To prevent peripheral muscarinic stimulation, scopolamine (Sigma–Aldrich) was injected subcutaneously at a dose of 1 mg/kg, 30 min before injection of pilocarpine (Liu et al., 2010). About 30 min after pilocarpine injection, animals became hypoactive and displayed oro-facial movements, salivation, eye blinking, twitching of vibrissae and yawning. Generalized seizure and limbic SE were observed 40–80 min after pilocarpine injection. Only mice that displayed 2-h of SE were selected in this study (Goffin et al., 2007; Curia et al., 2008). SE was stopped after 2-h with an injection of diazepam (10 mg/kg, Roche) in order to prevent mortality. SE initiated by pilocarpine injection triggers epileptogenesis which progress during about 1–2 weeks (Cavalheiro, 1995; Curia et al., 2008). Two hours after SE, animals were treated for 1 week with *P. daemia* extract, sodium valproate and vitamin C. During this period (i.e., the epileptogenesis period) mice were challenged with a convulsant. The challenge was characterized by the acute PTZ administration (Ilhan et al., 2005; Blanco et al., 2009). The challenge with PTZ was used to assess behaviorally the sensibility to a convulsant agent of animals treated or not with the plan extract (Ilhan et al., 2005; Blanco et al., 2009). Then, mice were placed in a 30 cm × 30 cm chambers for 30 min observation. A progressive evolution of seizure activity was evaluated using a six phase scale (Erakovic et al., 2001): (i) 0 indicated no response; (ii) 1, ear and facial twitching (iii) 2, convulsive waves axially through the body; (iv) 3, myoclonic body jerks; (v) 4 generalized clonic seizures turn over into side position; (vi) 5, generalized seizures with tonic extension episode and SE; and (vii) 6, death (Khalili et al., 2011). The latency and duration of the first myoclonic jerk, clonic seizure and generalized tonic–clonic seizure were measured. Latencies to generalized tonic–clonic seizure were used to calculate the seizure score as follows: S = 1 – (control latency/drug seizure latency) (Mehla et al., 2010).

##### Elevated plus-maze paradigm

Cognitive function was assessed using an elevated plus maze. The apparatus is made up of two open arms (16 cm × 5 cm) and two closed arms (16 cm × 5 cm × 10 cm) that extend from a common central platform (5 cm × 5 cm). The entire maze is elevated to a height of 50 cm above the floor level (Bum et al., 2011). Procedures were performed as previously described (Taiwe et al., 2015). Briefly, in the first task, each animal was placed at the end of the open arm and the initial transfer latency, i.e., the latency to closed arm entry was recorded. A 60 s cut-off was set. The mouse was then allowed to move freely in the maze for another 10 s (Taiwe et al., 2015). Similarly, 24-h later the latency to closed arm entry, termed retention transfer latency, was determined. Mice which did not enter the enclosed arm within 60 s on the second trial were assigned a score of 60 s (Taiwe et al., 2015).

##### T-maze paradigm

The T-shaped maze is made of wood and consists of a start arm and two choice arms. Each arm is 30 cm × 10 cm × 20 cm (length × width × height) (Taiwe et al., 2015). A recessed black plastic cup (3 cm in diameter, 1 cm in depth) containing food was placed on the floor at the end of each choice arm (Taiwe et al., 2015). A day before the experiment, each animal was placed in the start position (at the end of the start arm) for a 10 min exploration trial, one arm open and the other one closed and at the end, they were returned to their home cage. The following day, animals were reintroduced in the T-maze for a 5 min testing period (Taiwe et al., 2015). During the retrial (the two choice arms were opened), animals were placed in a start arm and the number of visits and the time spent in the two arms were recorded (Taiwe et al., 2015).

#### Biochemical Tests

Immediately after the animals were sacrificed, the brain hemispheres were quickly dissected out and cleaned with ice-cold saline (0.9%, w/v) to remove the hippocampus. After weighing the hippocampi, they were stored at -43°C. To perform biochemical analyses, 10% (w/v) homogenates prepared with ice-cold 0.1 M phosphate buffer (pH 7.4) were centrifuged (10,000 × *g*, 15 min). Aliquots of the supernatant were collected and used for biochemical estimation of reduced glutathione (GSH), protein, nitric oxide (NO), and malondialdehyde (MDA) levels. Superoxide dismutase (SOD) and catalase (CAT) activities were also determined from these tissues. Acetylcholinesterase (AchE) activity was assessed in hippocampi dissected from the right hemisphere (Mehla et al., 2010; Taiwe et al., 2015).

##### Total proteins quantification

The protein amount was estimated using the Biuret method (Gornall et al., 1949). The BSA (Carlsbad) was used as standard. Briefly, 3 ml of Biuret reagent (Sigma–Aldrich) and 10 μl of homogenate were added into test tubes. The contents were mixed by inversion and the absorbance was measured at 590 nm after 2 min against blank (3 ml of NaCl 0.9% mixed with 3 ml of Biuret reagent). The weight of protein was plotted against the corresponding absorbance resulting in a standard curve used to determine the protein in unknown samples. The concentration of protein was expressed in mg/ml of protein in the tissue.

##### AchE activity

The AchE activity was assessed by the Ellman method (Ellman et al., 1961). The assay mixture contained 0.05 ml of supernatant, 3 ml of sodium phosphate buffer (pH 8, Sigma–Aldrich), 0.1 ml of acetylthiocholine iodide (Sigma–Aldrich) and 0.1 ml of DNTB (Ellman reagent, Sigma–Aldrich). The change in absorbance was measured at 412 nm for 2 min, at 30 s intervals. Results were expressed in U/min/mg of protein in the tissue (1 U/min/mg of AchE was defined as the amount of enzyme that hydrolyzed 1 μmol of acetylthiocholine iodide).

##### SOD activity

The SOD activity in the tissues was determined by the method of Misra and Fridovich (1972), where the autoxidation of adrenaline (Sigma–Aldrich) is followed in terms of the production of adrenochrome (maximum absorption at 480 nm). Tissue homogenates (134 μl) were introduced in a test tube and 1666 μl of phosphate buffer (0.05 M, pH 10.2) in a blank tube to equilibrate the spectrophotometer. The reaction was started by adding 200 μl of freshly prepared adrenaline (0.3 mM). Then, the mixture was quickly mixed. The increase in absorbance at 480 nm was recorded at 20 and 80 s against the blank. One unit (U) of SOD was defined as the quantity of SOD required to inhibit 50% of the oxidation of adrenaline in adrenochrome for 1 min. The activity of SOD was expressed in U/min/mg of protein in the tissue.

##### CAT activity

The CAT activity was assayed following the method of Sinha (1972). In this method, dichromate (Sigma–Aldrich) in acetic acid (Sigma–Aldrich) is reduced to chromic acetate when heated in the presence of H_2_O_2_. The blue perchromic acid, an unstable intermediate is then formed. The reaction mixture consisted of 187.5 μl phosphate buffer (0.1 M, pH 7.5) and 12.5 μl of homogenate. The reaction was started by adding 50 μl of H_2_O_2_ (50 mM, Sigma–Aldrich). After 1 min, the reaction was stopped by the addition of 500 μl of dichromate acetic acid reagent. The tubes were immediately kept in a boiling water bath at 100°C for 10 min, and the green color developed during the reaction was read at 570 nm on a spectrophotometer against the blank. Blank tube, devoid of enzyme, was also processed in parallel. The amount of H_2_O_2_ remaining was determined using a standard curve. The enzyme activity was expressed in mmol of H_2_O_2_ consumed/min/mg of protein in brain tissue. The specific activity of CAT was calculated as follows: CAT activity = [(*A* of sample – *A* of blank) × *f*/(*a* × *t* × *mi*)]. Where *A* is the absorbance, *f* the dilution factor, *a* standard curve coefficient, *t* the time in minute and *mi* the weight of tissue processed.

##### GSH level

Glutathione was measured using the method of Ellman (1959). Briefly, 1500 μl of DNTB and 500 μl of Tris-HCl (Sigma–Aldrich) buffer (50 mM, pH 7.4) were added to a blank tube containing 100 μl of Tris-HCl buffer (50 mM, pH 7.4) or to test tubes containing tissue homogenates (100 μl). The mixture solution was incubated for 1 h, and the absorbance was read against the blank at 412 nm. The GSH concentration was calculated using an extinction coefficient of 13600 mol^-1^cm^-1^. The concentration of GSH was expressed as μmol/g of protein in the tissue.

##### MDA level

The method of Wilbur et al. (1949) for MDA determination was used. Briefly, distilled water (250 μl) and homogenate (20 μl) were introduced in the control tube and in the test tubes, respectively. Then, 250 μl of Tris-HCl buffer (50 mM, pH 7.4), 500 μl of trichloroacetic acid (20%, Sigma–Aldrich) and 1000 μl of thiobarbituric acid (0.67%, Sigma–Aldrich) were added. The mixture solution was heated in a water-bath (90°C, 10 min). After cooling at room temperature, the tubes were centrifuged (3000 rpm, 15 min). The absorbance of the pink-colored supernatant was measured against the blank at 530 nm. The MDA concentration was calculated using an extinction coefficient of 1.56 × 10^5^ mmol^-1^cm^-1^. MDA level was expressed in μmol/g of protein in the tissue.

##### NO level

Nitric oxide content was assayed by the Griess method (Grand et al., 2001). NO is a compound with a short half-life that is rapidly converted to the stable end products nitrate (NO_3_^-^) and nitrite (NO_2_^-^). In this assay, the conversion of nitrate into nitrite is accompanied by color development in the presence of [0.1% *N*-(1-naphthyl) ethylenediamine dihydrochloride, 1% sulfanilamide and 2.5% phosphoric acid) in acidic medium] (Griess reagent, Sigma–Aldrich) (Grand et al., 2001). To estimate the quantity of NO, 200 μl of homogenate and 200 μl of Griess reagent were introduced in test tubes. The solution was mixed and the absorbance was read at 570 nm after 10 min. A standard curve NaNO_2_ was established with a set of serial dilutions of nitrite. Linear regression was done by using the peak area from nitrite standard. The resulting equation was used to calculate the unknown sample concentrations. Results were expressed in mmol/g of protein in the tissue.

### *In Vitro* Studies

Primary cortical neuron cultures were prepared from the cerebral cortex of Wistar rat embryo of 17 days as described previously (Kim et al., 1998). Briefly, pregnant Wistar rats were anesthetized with sodium pentobarbital (30 mg/kg, i.p., Sigma–Aldrich) and sacrificed by cervical dislocation. The cerebral cortex of fetal rats was rapidly removed bilaterally and collected. Tissues were then gently minced using a sterile razor blade and digested in PBS (0.1 M, pH 7.4, Sigma–Aldrich) for 15 min. A Pasteur pipette was used for dissociation of cells (approximately 5–10 times). After centrifugation (200 × *g* for 3 min), cells were re-suspended in DMEM (Sigma–Aldrich) supplemented with FBS (15%, Carlsbad), L-glutamine (2 mM, Sigma–Aldrich), sodium bicarbonate (4.2 mM, Sigma–Aldrich), BSA (0.3 g/l, Sigma–Aldrich), β-mercaptoethanol (0.1 mM, Sigma–Aldrich), penicillin (1%, Sanofi Aventis), streptomycin (50 μg/ml, Sanofi Aventis) and grown on 0.1% poly-L-Lysine (Sigma–Aldrich) coated plates. Cultures were incubated at 37°C in a humidified 5% CO_2_ atmosphere. To prevent proliferation of non-neuronal cells, cytosine β-D-arabinofuranoside hydrochloride (10 μM, Sigma–Aldrich) was added 3 days after plating. In all experiments, 11 days mature cells were used.

#### Cell Viability Assay by MTT

3-(4,5-Dimethylthiazol-2-yl)-2,5-diphenyltetrazolium bromide test is based on the ability of viable cells to metabolize a tetrazolium salt to formazan blue in the mitochondria (Loveland et al., 1992). The formazan accumulation is proportional to the number of viable cells and inversely proportional to the degree of cytotoxicity (Berridge et al., 2005). Briefly, sample supernatants are inoculated in 96 wells. Cortical cell cultures were treated and incubated with 10 μl of aqueous extract of *P. daemia* (5, 10, 19, 40, 77, 153, 306, 615, 1225, 2450 μg/ml) for 1 h. Cultures were then exposed to L-glutamate (10 mM) and maintained for 24 h (Kim et al., 1998). After the incubation, culture medium was removed before adding 100 μl of solution of MTT (1 mg/ml, Sigma–Aldrich). The plates were incubated during 1 h at 37°C. Excess MTT was removed and 100 μl of DMSO (0.1%, Sigma–Aldrich) were added to each well to dissolve formazan crystals (precipitates resulting from the conversion of MTT by the mitochondrial succinate dehydrogenase). The plates were vortexed for 5 min and read at 540 nm with a microplate reader. The percentage of cell viability was expressed according to the following formula: percentage of cell viability = 100 × [(optical density (OD) of L-glutamate + extract treated cultures) - (OD of L-glutamate treated cultures)/(OD of control cultures - OD of L-glutamate treated cultures)] (Koo et al., 2006).

#### Cell Viability Assay by Lactate Dehydrogenase (LDH)

Lactate dehydrogenase (LDH) is a cytosolic enzyme present in many different cell types. Plasma membrane damage releases LDH into the cell culture media (Decker and Lohmann-Matthes, 1988). Extracellular LDH in the media can be quantified by a coupled enzymatic reaction in which LDH catalyzes the conversion of lactate to pyruvate via NAD^+^ reduction to NADH (Decker and Lohmann-Matthes, 1988). Therefore, NADH is used to reduce a tetrazolium salt to a red formazan product that can be measured at 490 nm (Nachlas et al., 1960). For the assay 20 μl of lactate solution (36 mg/ml of 10 mM Tris buffer, pH 8.5, Sigma–Aldrich) were added to the samples in microliter wells, followed by 20 μl solution of MTT (2 mg/ml of PBS (0.1 M, pH 7.4) prepared from a 10-fold concentrated stock solution in DMSO). The enzymatic reaction was then started by addition of 20 μl of a solution containing NAD^+^ (3 mg/ml, Sigma–Aldrich) and diaphorase (13.5 U/ml; BSA: 0.03%; sucrose: 1.2%; in PBS, Sigma–Aldrich) and allowed to proceed for 20 min (Decker and Lohmann-Matthes, 1988). The reaction was terminated by the addition of 20 μl of the LDH inhibitor oxamate (16.6 mg/ml of PBS, Sigma–Aldrich). Measurements were performed at 490 nm with a microplate reader. Percentage of cell viability was evaluated as above.

### Quantification of Apoptosis and Necrosis by Hoechst 33342 and Propidium Iodide Staining

The experiment was realized according to the method described by Syed et al. (2013). Briefly, cells were grown in tissue culture dishes and treated with or without the aqueous extract of *P. daemia* at concentrations (5, 10, 19, 40, 77, 153, 306, 615, 1225, 2450 μg/ml). After 24 h of incubation in an incubator (37°C in 5% CO2), the cells were harvested and washed with cold PBS (0.1 M, pH 7.4). The cells were suspended in Hoechst 33342 solution (10 μg/ml, Sigma–Aldrich) and were incubated (37°C in 5% CO_2_) for 7 min (Syed et al., 2013). After incubation with Hoechst 33342, the cells were stained with propidium iodide (2.5 μg/ml, Sigma–Aldrich). The samples were maintained in the dark for 15 min. After staining, an aliquot of cell suspension was placed on a glass microscope slide. The slides were observed immediately under a fluorescence microscope and the fluorescence was measured at 630× magnification. Cells were counted and the numbers of each of the four cellular states were recorded and analyzed using fluorescence microscopy for quantification of apoptosis and necrosis (Moongkarndi et al., 2004; Syed et al., 2013). The experiment was conducted in triplicates. Hoechst 33342 was used to determine apoptotic nuclear morphology, while propidium iodide indicated dead cells by necrosis. Cells with fragmented or condensed nuclei were considered as apoptotic cells. After the exclusion of the positive apoptotic cells from Hoechst 33342, the propidium iodide positive cells were considered necrotic cells (Moongkarndi et al., 2004). The numbers of apoptotic or necrotic cells in the treatment groups were compared to the control. The percentages of apoptotic and necrotic cells were determined according to the following formula:

– Percentage of apoptotic cells = [(LA + DA)/LN + LA + DN + DA) × 100];
– Percentage of necrotic cells = [(DN/LN + LA + DN + DA) × 100] (Brady, 2004; Syed et al., 2013). Where LN are live target cells with normal nuclei (Hoechst 33342/propidium iodide: blue chromatin with organized structure), LA are live cells with apoptotic nuclei (Hoechst 33342/propidium iodide: bright blue chromatin that is highly condensed or fragmented), DN are dead cells with normal nuclei (Hoechst 33342/propidium iodide: pink chromatin with organized structure), and DA are dead cells with apoptotic nuclei (Hoechst 33342/propidium iodide: bright pink chromatin that is highly condensed or fragmented).

### Statistical Analysis

Inter-group differences were assessed using one-way analysis of variance (ANOVA), followed by Newman Keul’s multiple comparisons *post hoc* test. The significance level was set at *p* < 0.05, with Mann–Whitney *U* test correction. Analyses were performed using Graph Pad Prism version 5.1 for Windows (Graph Pad Software, San Diego, CA, United States) and XLSTAT, 2007. Data were expressed as mean ± standard error of the mean (SEM) for *in vivo* tests and as percentage for *in vitro* tests.

## Results

### Effects of *P. daemia* on Seizures Induced by PTZ Challenge

#### Latency to Seizures

**FIGURE 1:**
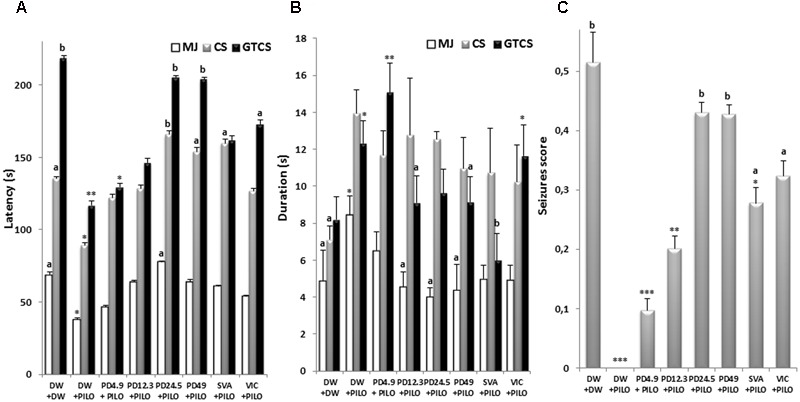
Effect s of *P. daemia* on PTZ challenge outcome. **(A)** Effects of *P. daemia* extract on the latency to myoclonic jerks, clonic seizures and generalized tonic–clonic seizures induced by pentylenetetrazol challenge in pilocarpine-injected mice. **(B)** Effect of *P. daemia* extract on the duration of myoclonic jerks, clonic seizures and generalized tonic–clonic seizures in the same animals. **(C)** Effects of *P. daemia* on the seizures score. Data are mean ± SEM, *N* = 7 per group. Newman Keul’s multiple comparisons *post hoc* test, with Mann–Whitney *U* test correction: (i) vs. control animals (DW + DW group) receiving only distilled water: ^∗^*p* < 0.05, ^∗∗^*p* < 0.01; (ii) ^∗∗∗^*p* < 0.001 vs. disease control animals (DW + PILO group) receiving distilled water and pilocarpine: ^a^*p* < 0.05, ^b^*p* < 0.01. CS, clonic seizures; DW, distilled water; MJ, myoclonic jerks; GTCS, generalized tonic–clonic seizures; PD, *Pergularia daemia*; PILO, pilocarpine; VIC, vitamin C; SVA, sodium valproate.

The mice treated with PTZ resulted in a classical pattern of limbic motor seizures culminating into generalized tonic–clonic seizures. A decreased myoclonic jerks latency was observed in DW + PILO group compared to DW + DW group (*p* < 0.05) (**Figure 1A**). *P. daemia* caused a two-fold increase (*p* < 0.05) in the latency to myoclonic jerks compared to DW + PILO group (37.87 ± 1.33 s in DW + PILO group against 77.8 ± 2.18 s in the group administered with *P. daemia* dose 24.5 mg/kg) [*F*(7,49) = 23.00, *p* < 0.0001] (**Figure 1A**). Sodium valproate induced an increase of this latency which did not reach statistical significance (**Figure 1A**).

Latency to clonic seizures was decreased in DW + PILO group compared to DW + DW group (*p* < 0.05) (**Figure 1A**). *P. daemia* increased the latency to clonic seizures compared to DW + PILO group [*F*(7,49) = 121.25, *p* < 0.001] (**Figure 1A**). The latency to clonic seizure increased and reached 165.97 ± 2.38 s in the group administered with *P. daemia* dose 24.5 mg/kg (*p* < 0.01) (**Figure 1A**). This effect was stronger than sodium valproate effect (159.92 ± 2.42 s, *p* < 0.05) (**Figure 1A**).

Similarly, a decreased generalized tonic–clonic seizure latency was observed in DW + PILO group compared to DW + DW group (*p* < 0.01) (**Figure 1A**). *P. daemia* (24.5 mg/kg) increased significantly and the latency to generalized tonic–clonic seizures [*F*(7,49) = 312.14, *p* < 0.001], compared to DW + PILO group in a dose dependent manner up to 205.08 ± 1.25 s (*p* < 0.01) in the group administered with *P. daemia* dose 24.5 mg/kg. This effect was stronger than sodium valproate (162.10 ± 2.97 s, *p* > 0.05) and vitamin C (173.12 ± 2.89 s, *p* < 0.05) effect (**Figure 1A**).

#### Seizure Duration

Significant inter-group differences were observed in the duration of myoclonic jerks [*F*(7,49) = 1.33, *p* < 0.01], clonic seizures [*F*(7,49) = 2.45, *p* < 0.001] and generalized tonic–clonic seizures [*F*(7,49) = 7.66, *p* < 0.0001]. *P. daemia* decreased the duration of myoclonic jerks from 8.44 ± 1.03 s in DW + PILO group to 4.00 ± 0.48 s (*p* < 0.05) in the group administered with *P. daemia* dose 24.5 mg/kg (**Figure 1B**). The duration was slightly decreased from 8.44 ± 1.03 s in DW + PILO group to 4.98 ± 0.73 s (*p* > 0.05), 4.94 ± 0.80 s (*p* > 0.05) in the groups administered with sodium valproate and vitamin C, respectively (**Figure 1B**). Generalized tonic–clonic seizures duration was decreased from 12.30 ± 1.26 s in DW + PILO group to 9.09 ± 1.30 s (*p* < 0.05) and 9.14 ± 1.36 s (*p* < 0.05) in the groups administered with *P. daemia* doses 12.3 and 49 mg/kg, respectively (**Figure 1B**).

#### Seizure Score

A significant reduction in seizures score was observed in DW + PILO group compared to DW + DW group (*p* < 0.01) (**Figure 1C**). Overall, seizures score between the groups was also significantly different [*F*(7,49) = 73.11, *p* < 0.001]. *P. daemia* increased the seizures score from 0 in DW + PILO group to 0.43 ± 0.02 (*p* < 0.01) and 0.43 ± 0.02 (*p* < 0.01), respectively, in groups treated with *P. daemia* doses 24.5 and 49 mg/kg (**Figure 1C**). The seizures score was also increased in groups treated with sodium valproate (0.28 ± 0.03, *p* < 0.05) and vitamin C (0.32 ± 0.03, *p* < 0.05) (**Figure 1C**).

### Effects of *P. daemia* on Memory

#### Elevated Plus Maze

**FIGURE 2:**
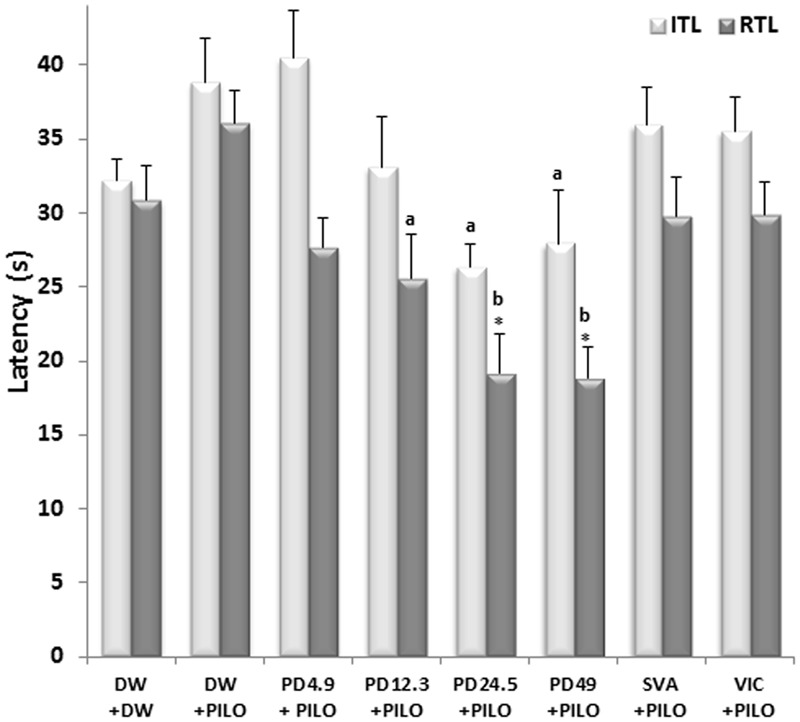
Effects of *P. daemia* on elevated plus maze parameters. Data are mean ± SEM, *N* = 7 per group. Newman Keul’s multiple comparisons *post hoc* test, with Mann–Whitney *U* test correction: (i) vs. control animals (DW + DW group) receiving only distilled water: ^∗^*p* < 0.05; (ii) vs. disease control animals (DW + PILO group) receiving distilled water and pilocarpine: ^a^*p* < 0.05, ^b^*p* < 0.01. DW, distilled water; PD, *Pergularia daemia*; PILO, pilocarpine; VIC, vitamin C; SVA, sodium valproate; ITL, initial transfer latency; RTL, retention transfer latency.

Significant inter-group differences were observed in the initial transfer latency in the elevated plus maze [*F*(7,49) = 6.04, *p* < 0.001]. *P. daemia* decreased initial transfer latency up to 26.35 ± 1.56 s at dose 24.5 mg/kg, against 39.84 ± 2.60 s in DW + PILO group (*p* < 0.05) (**Figure 2**). Intergroup differences were observed in the retention transfer latency [*F*(7,49) = 5.99, *p* < 0.01]. *P. daemia* induced dose-dependent decrease from 36.03 ± 2.13 s in DW + PILO group to 18.84 ± 2.05 s (*p* < 0.01) in the group administered with *P. daemia* dose 49 mg/kg (**Figure 2**). However, sodium valproate and vitamin C did not induce significant decreases in initial and retention transfer latencies (**Figure 2**).

#### T-maze

**FIGURE 3:**
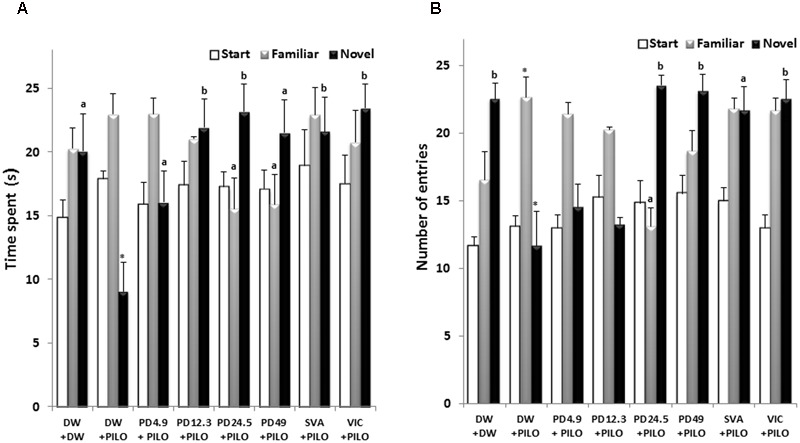
Effects of *P. daemia* on T-maze parameters. **(A)** Effects of *P. daemia* on the time spent in the start, novel and familiar T-maze. **(B)** Effect of *P. daemia* on the number of entries in start, novel and familiar T-maze arms. Data are mean ± SEM, *N* = 7 per group. Newman Keul’s multiple comparisons *post hoc* test, with Mann–Whitney *U* test correction: (i) vs. control animals (DW + DW group) receiving only distilled water: ^∗^*p* < 0.05; (ii) vs. disease control animals (DW + PILO group) receiving distilled water and pilocarpine: ^a^*p* < 0.05, ^b^*p* < 0.01. DW, distilled water; PD, *Pergularia daemia*; PILO, pilocarpine; VIC, vitamin C; SVA, sodium valproate.

No inter-group difference was observed in the number of entries in the start arm (**Figure 3B**). *P. daemia* decreased the time spent in the familiar arm (14.92 ± 2.30 s at dose 24.5 mg/kg vs. 22.90 ± 1.44 s in DW + PILO group, *p* < 0.05) (**Figure 3A**). As sodium valproate and vitamin C, *P. daemia* increased the time spent in the novel arm up to 23.16 ± 2.16 s at dose 24.5 mg/kg (against 9.08 ± 2.24 s in DW + PILO group, *p* < 0.01) (**Figure 3A**).

Similar, no inter-group difference was observed in the number of entries in the start arm (**Figure 3B**). The number of entries in the familiar arm was decreased up to 13.14 ± 1.35 in the group treated with *P. daemia* dose 24.5 mg/kg (against 22.71 ± 1.48 in DW + PILO group, *p* < 0.05) (**Figure 3B**). Conversely, the number of entries in novel arm was increased up to 23.57 ± 0.72 (*p* < 0.01) and 23.14 ± 1.22 (*p* < 0.01) in groups treated with *P. daemia* doses 24.5 and 49 mg/kg, respectively (against 11.71 ± 2.53 in DW + PILO group) (**Figure 3B**). Although in a lesser extent, sodium valproate and vitamin C also increased the number of entries in the novel arm (**Figure 3B**).

### Levels of Total Proteins, AchE, Antioxidant Enzymes, and Oxidative Stress Markers

#### Total Level of Protein

**FIGURE 4:**
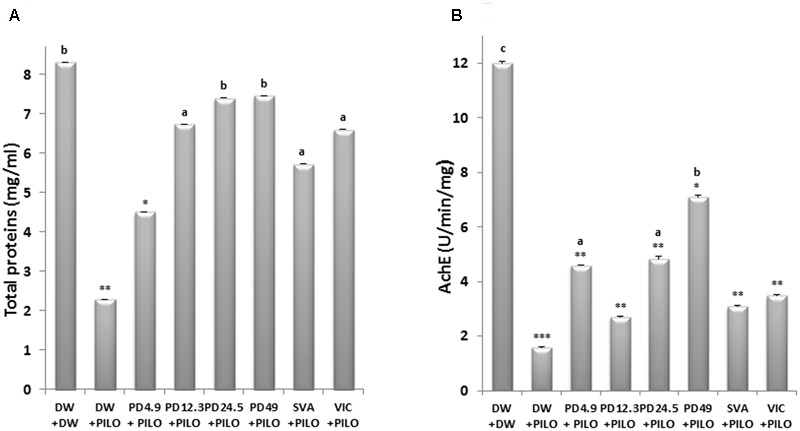
Effects of *P. daemia* on total proteins level and AchE activity. **(A)** Effects of *P. daemia* on total proteins level in hippocampi of pilocarpine-injected mice. **(B)** Effect of *P. daemia* on AchE level in hippocampi of the same animals. Data are mean ± SEM, *N* = 7 per group. Newman Keul’s multiple comparisons *post hoc* test, with Mann–Whitney *U* test correction : (i) vs. control animals (DW + DW group) receiving only distilled water: ^∗^*p* < 0.05, ^∗∗^*p* < 0.01, ^∗∗∗^*p* < 0.001; (ii) vs. disease control animals (DW + PILO group) receiving distilled water and pilocarpine: ^a^*p* < 0.05, ^b^*p* < 0.01. DW, distilled water; PD, *Pergularia daemia*; PILO, pilocarpine; VIC, vitamin C; SVA, sodium valproate.

Significant inter-group differences were observed in hippocampus total proteins level [*F*(7,49) = 130.20, *p* < 0.001]. Pilocarpine significantly decreased the protein level up to 2.29 ± 0.00 mg/ml wet tissue in DW + PILO group, against 8.32 ± 0.00 mg/ml in the DW + DW group (*p* < 0.01) (**Figure 4A**). Treatment with *P. daemia* prevented such decrease in a dose-dependent manner. At dose 49 mg/kg, the extract resulted in protein level comparable to DW + DW group (7.45 ± 0.00, *p* < 0.01 vs. DW + PILO group) (**Figure 4A**). The well-established antioxidant vitamin C also prevented pilocarpine-induced protein decrease (6.60 ± 0.00, *p* < 0.05 vs. DW + PILO group) (**Figure 4A**).

#### AchE Activity

Significant inter-group differences were observed in AchE activity [*F*(7,49) = 244.76, *p* < 0.001]. Pilocarpine decreased AchE activity compared to DW + DW group (1.59 ± 0.00 U/min/mg in DW + PILO group vs. 12.01 ± 0.00 U/min/mg, *p* < 0.001) (**Figure 4B**). *P. daemia* dose 49 mg/kg prevented the decrease in AchE activity caused by pilocarpine (7.10 ± 0.00 U/min/mg, *p* < 0.01 vs. DW + PILO group) (**Figure 4B**).

#### Antioxidant Enzymes

The effects of *P. daemia* extract on activities of the antioxidant enzymes tested (CAT and SOD) in hippocampi of pilocarpine-injected mice is shown in **Table 1**. *P. daemia* treatment increased SOD (*p* < 0.05) and CAT (*p* < 0.01) activities compared to DW + PILO group. Vitamin C also increased CAT activity. Although to a lesser extent, sodium valproate also displayed some of these effects, particularly the marked decrease in CAT activity induced by pilocarpine (**Table 1**).

**Table 1:** Effects of *P. daemia* on antioxidant enzymes and oxidative stress markers in hippocampi of pilocarpine-injected mice.

Treatment	Dose (mg/kg)	MDA (μmol/g)	GSH (μmol/g)	SOD (U/min/mg)	CAT (mmol H_2_O_2_/min/mg)	NO (mmol/g)
DW + DW	– + –	0.17 ± 0.00^b^	11.67 ± 0.54^a^	14.71 ± 0.11^a^	59.26 ± 0.39^a^	0.17 ± 0.00
DW + PILO	– + 360	0.51 ± 0.01^∗∗^	4.41 ± 0.02^∗^	12.32 ± 0.18^∗^	37.32 ± 0.20^∗^	0.18 ± 0.00
PD + PILO	4.9 + 360	0.46 ± 0.00^∗∗^	7.91 ± 0.05^∗^	12.88 ± 0.10	36.37 ± 1.70^∗^	0.17 ± 0.00
PD + PILO	12.3 + 360	0.44 ± 0.01^∗∗^	10.61 ± 0.08	12.71 ± 0.17	48.38 ± 0.38^a^	0.19 ± 0.10
PD + PILO	24.5 + 360	0.21 ± 0.00^∗a^	10.60 ± 0.09	15.79 ± 0.10^a^	77.29 ± 0.26^∗b^	0.18 ± 0.02
PD + PILO	49 + 360	0.28 ± 0.01^∗a^	11.35 ± 0.13^a^	16.87 ± 0.03^∗a^	107.80 ± 0.49^∗∗b^	0.26 ± 0.12^∗a^
SVA + PILO	300 + 360	0.42 ± 0.01^∗∗^	7.86 ± 0.03^∗^	12.05 ± 0.14^∗^	59.42 ± 0.38^a^	0.27 ± 0.05^∗a^
VIC + PILO	250 + 360	0.36 ± 0.01^∗^	9.36 ± 0.12	14.41 ± 0.21^a^	102.18 ± 0.91^∗∗b^	0.13 ± 0.04

*Data are mean ± SEM, *N* = 7 per group. Newman Keul’s multiple comparisons *post hoc* test, with Mann–Whitney *U* test correction: (i) vs. control animals (DW + DW group) receiving only distilled water: ^∗^*p* < 0.05, ^∗∗^*p* < 0.01, (ii) vs. disease control animals (DW + PILO group) receiving distilled water and pilocarpine: 0.05 ^a^*p* < 0.05, ^b^*p* < 0.01. DW, distilled water; PD, *Pergularia daemia*; PILO, pilocarpine; VIC, vitamin C; SVA, sodium valproate; MDA, malondialdehyde; GSH, reduced glutathione; SOD, superoxide dismutase; CAT, catalase; NO, nitric oxide.*

#### Oxidative Stress Markers Level

The effects of *P. daemia* extract on levels of oxidative stress markers tested (GSH, MDA, and NO) in hippocampi of pilocarpine-injected mice is shown in **Table 1**. *P. daemia* treatment induced moderate increase (*p* < 0.05) in the GSH level compared to DW + PILO group. On the other hand, the extract decreased significantly the MDA level (*p* < 0.05) and, surprisingly, increased the estimated NO level. Although to a lesser extent, sodium valproate also displayed a non-significant decrease in GSH level and MDA level induced by pilocarpine (**Table 1**).

### *In Vitro* Neuroprotective Effects of *P. daemia*

#### Protective Effect of *P. daemia* Extract against L-Glutamate-Induced Neurotoxicity

**FIGURE 5:**
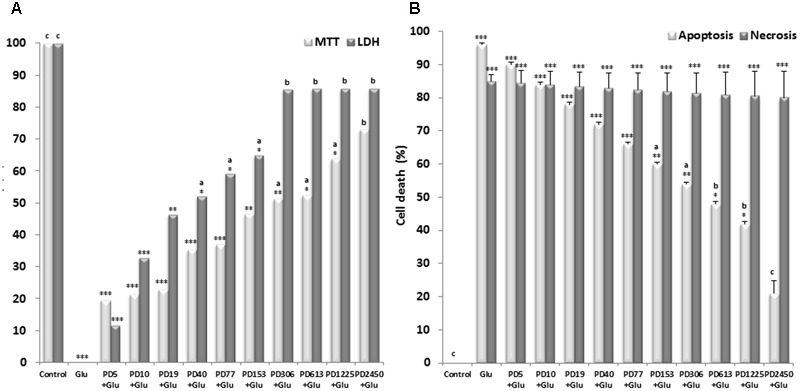
Effects of *P. daemia* against L-glutamate-induced neurotoxicity, apoptosis and necrosis. **(A)** Effects of *P. daemia* against L-glutamate-induced neurotoxicity. **(B)** Effects of *P. daemia* against L-glutamate-induced apoptosis and necrosis. Quantitative analysis of the histograms expressed as the percentage of cell viability or cell death by apoptosis and necrosis. Data are mean ± SEM. Newman Keul’s multiple comparisons *post hoc* test, with Mann–Whitney *U* test correction : (i) vs. control: ^∗^*p* < 0.05, ^∗∗^*p* < 0.01, ^∗∗∗^*p* < 0.001; (ii) vs. L-glutamate : ^a^*p* < 0.05, ^b^*p* < 0.01, ^c^*p* < 0.001. PD, *Pergularia daemia* (5, 10, 19, 40, 77, 153, 306, 615, 1225, 2450 μg/ml); MTT, 3-(4,5-dimethylthiazol-2-yl)-2,5-diphenyl tetrazolium bromide; LDH, lactate dehydrogenase; Glu, L-glutamate (10 mM); PD, *Pergularia daemia* (5, 10, 19, 40, 77, 153, 306, 615, 1225, 2450 μg/ml).

In the MTT test, stimulation with L-glutamate alone resulted in a decrease in cell viability up to approximately 0.19% compared to control. Nevertheless, the different doses of the extract exhibited a significant decrease of L-glutamate-induced toxicity in a dose dependent manner. The highest concentration of the extract exhibited protective effect (85.92% vs. control) (**Figure 5A**). The protective effect of *P. daemia* was also revealed by LDH release assay. As shown in **Figure 5A**, cell viability decreased up to approximately 0.39% after exposure to L-glutamate. However, treatment with *P. daemia* resulted in a significant increase of this viability up to 73.01% at the highest concentration.

#### Protective Effect of *P. daemia* against L-Glutamate-Induced Apoptosis and Necrosis

Results of Hoechst staining in control culture indicated that, after exposure to L-glutamate, cortical neurons exhibited high levels of condensed chromatin and apoptotic bodies, indicating an increase of apoptotic cells up to 93.00% (**Figure 5B**). Treatment with *P. daemia* resulted in a significant decrease of these apoptotic features up to 19.67% at the highest concentration (**Figure 5B**). Results of propidium iodine staining in control culture indicated that, after exposure to L-glutamate, cortical neurons culture exhibited high levels of degenerated neurons, indicating an increase of necrotic cells up to 89.67% (**Figure 5B**). *P. daemia* was not able to protect neurons against L-glutamate induced cell necrosis (**Figure 5B**).

## Discussion

The aim of this study was to evaluate the antiepileptogenic and neuroprotective effects of the decoction of *P. daemia* roots. *In vivo* and *in vitro* experimental models were used. As the results show, the acute administration of PTZ in mice treated with distilled water (DW + PILO group) for 1 week (epileptogenesis period) after SE, induced an increase in the latency of seizures, and a decrease in the duration and score of seizures (Blanco et al., 2009). Remarkably, our results demonstrate that PTZ produces different effects when injected in epileptogenic and non-epileptogenic mice. This is a significant demonstration that the pharmacologic response outline of acute seizures contrasts from that of chronic seizures paradigms (Loscher et al., 1991; Blanco et al., 2009). In the present study, *P. daemia* reduced the severity of seizures induced by PTZ challenge on epileptogenic process. *P. daemia* extract also increased the seizure score dose dependently. Such reduction in seizure severity and in seizure susceptibility to a convulsant during epileptogenesis process suggests that the decoction antagonized or altered the epileptogenic process induced by pilocarpine (Pitkanen et al., 2005; Mehla et al., 2010; Pitkanen, 2010). Indeed, the PTZ (GABA_A_ receptor complex antagonist) is known to increase the seizure threshold and therefore to induce more severe seizures in epileptogenic brain compared to non-epileptogenic brain (Ilhan et al., 2005; Blanco et al., 2009; Mehla et al., 2010; Taiwe et al., 2015). Thus, the challenge is used to test behaviorally the susceptibility to a convulsant agent with or without treatment with the plant extract (Ilhan et al., 2005; Blanco et al., 2009). The findings of the present study suggest therefore that the aqueous extract of *P. daemia* has antiepileptogenic effects in mouse model of TLE. The effects of *P. daemia* on seizures induced by PTZ were more marked than those of vitamin C (powerful antioxidant), which was previously reported to mitigate epileptogenesis by blocking the efflux, rather than influx, of calcium, and therefore it interferes with these mechanisms (Xavier et al., 2007; dos Santos et al., 2011). These observations suggest that the extract of *P. daemia* could interfere with the mechanisms of neurotransmitter release and/or uptake from neuronal terminals. The effects of *P. daemia* were also more marked than those of the antiepileptic drug sodium valproate. The main mechanisms of valproate include an increase in GABAergic activity, reduction in excitatory neurotransmission and modification of monoamines (Xavier et al., 2007; Loscher and Brandt, 2010; Rahmati et al., 2013; Taiwe et al., 2015). These observations suggest that *P. daemia* could have altered the epileptogenesis process by increasing GABAergic activity and by reducing excitatory neurotransmission. The antioxidant and anticonvulsant effects of the plant probably mediated by different molecules or mechanisms could have as result a synergic effect greater than the effect of Vitamin C or sodium valproate alone.

Furthermore, *P. daemia* extract improved cognitive processes as revealed by the elevated plus maze and T-Maze. Considering that cognitive impairment or decline can also be associated with epileptogenesis in TLE (Cha et al., 2002; Stafstrom, 2006; Kumar et al., 2008; Mehla et al., 2010; Pahuja et al., 2013), these findings further suggest that *P. daemia* extract has antiepileptogenic effects.

The loss of neurons in the hippocampus is the first event characterizing epileptogenesis. This loss of neurons is associated with a significant decrease in total proteins (Dalby and Mody, 2001; Yamamoto and Mohanan, 2003; Patsoukis et al., 2005; Waldbaum and Patel, 2010). Given that *P. daemia* prevented the decrease in total proteins induced by pilocarpine, these results suggest that *P. daemia* has neuroprotective effects (Bahndari et al., 2008; Waldbaum and Patel, 2010). Interestingly, the drastic decrease in AchE activity, marker of neuronal loss (Cavazos and Sutula, 1990; Duysen et al., 2002; Veerendra and Gupta, 2002; Freitas et al., 2005; Niessen et al., 2005), was prevented by *P. daemia*. These results also suggest that *P. daemia* has neuroprotective effects. In addition, these effects were more marked than those of vitamin C and valproate sodium. These drugs are known to prevent neuronal loss by preventing oxidative stress (Xavier et al., 2007; dos Santos et al., 2011) and by increasing GABA neurotransmission (Brandt et al., 2003; Loscher and Brandt, 2010), respectively. Altogether, these observations suggest that *P. daemia* antiepileptogenic effects are mediated by neuroprotective effects.

To confirm the implication of antioxidant pathways in the realization of antiepileptogenic effects of *P. daemia*, the effects of the extract on antioxidant enzymes and oxidative stress markers in the hippocampus were assessed. In the present study, the activity of SOD enzyme, which protects cells against harmful superoxide radicals and the resulting oxidative stress (Agarwal et al., 2011; Shin et al., 2011), was drastically increased in groups receiving the extract. The activity of CAT enzyme, which eliminate H_2_O_2_ and its toxic radicals resulting from the antioxidant action of SOD (Freitas et al., 2005; Karthishwaran and Mirunalini, 2012; Kiasalari et al., 2013), was also increased in groups treated with *P. daemia*, in dose dependent manner. These results suggest that the extract induced its antioxidant activities by increasing SOD and CAT activities (Karthishwaran and Mirunalini, 2012; Kiasalari et al., 2013).

Furthermore, decrease in level of MDA, a lipid peroxidation marker caused by free radicals (Dal-Pizzol et al., 2000; Ilhan et al., 2005; Balaji et al., 2013), was also observed. *P. daemia* treatment increased significantly the tissue levels of GSH, an endogenous antioxidant that reacts with free radicals and prevents the generation of hydroxyl radical (Schulz et al., 2000; Gupta et al., 2003; Agarwal et al., 2011). Altogether, these results also suggest that *P. daemia* antioxidant activity is mediated in part by the decrease in the MDA level and by the increase in the GSH level. These results are in agreement with a report by Bhaskar and Balakrishnan (2009) in which *P. daemia* decreased the MDA level and increased the GSH level. However, the level of NO, whose radicals cause oxidative damage via lipid peroxidation (Ilhan et al., 2005; Khadrawy et al., 2013), was not improved in the present study. This result suggests that *P. daemia* antioxidant activity is not mediated by the inhibition of NO production. This result is in agreement with the study reported by Balaji et al. (2013). Overall, changes in the levels of antioxidant enzymes and oxidative stress markers observed strongly suggest that *P. daemia* extract has antioxidant properties. These properties were more effective than those of vitamin C, a powerful antioxidant known to increase the SOD and CAT activities, and to decrease the MDA level by scavenging free radicals (Xavier et al., 2007; dos Santos et al., 2011). Taken together, these results suggest that *P. daemia* antiepileptogenic effects are partly mediated by its antioxidant properties.

Neuronal cell death is a pathophysiological consequence of many brain insults that induced epilepsy (Henshall and Engel, 2013). This event is implicated as a causal factor in epileptogenesis (Henshall and Engel, 2013). Overactivation of glutamate receptors under pathophysiological conditions leads to excitotoxic cell death (Meldrum, 2002; Brown and Bal-Price, 2003). The present findings show that *P. daemia* significantly protected cortical neurons against excitotoxicity induced by L-glutamate. These results suggest that *P. daemia* has neuroprotective effects mediated in part by antiapoptotic mechanisms (Narkilahti et al., 2003; Gandhi and Abramov, 2012). Thus, these results explain and confirm antiepileptogenic and neuroprotective effects of *P. daemia* extract *in vivo*.

## Conclusion

In this study, we investigated the antiepileptogenic and neuroprotective effects of aqueous extract of *P. daemia* using *in vivo* and *in vitro* approaches. In *in vivo* studies, oral administration of the extract resulted in reduction in the severity of seizures and cognitive impairment. The study of AchE activity and oxidative stress markers revealed that *P. daemia* extract may mediate its antiepileptogenic effects at least partly through its antioxidant properties. In *in vitro* studies *P. daemia* protected cells against death induced by L-glutamate. This effect may be mediated by antiapoptotic pathways. Taken together, these findings indicate that *P. daemia* has antiepileptogenic and neuroprotective effects. Future experiments aimed at characterizing further the antiepileptogenic properties of *P. daemia* extract should be designed, considering the therapeutic potential for TLE. This plant could exert some beneficial effect in the threshold of seizures and could be used as complementary treatment for epilepsy and other neurological diseases.

## Author Contributions

AK, SP, and GT performed all behavioral studies, accomplished the data analysis and drafted the manuscript. AK, EN, SP, and GT designed the study. EN critically revised the manuscript for important intellectual content. FM, GNg, GNk, JN, JO, SP, and NK helped in *in vivo* studies. All authors have read and approved the final manuscript.

## Conflict of Interest Statement

The authors declare that the research was conducted in the absence of any commercial or financial relationships that could be construed as a potential conflict of interest.
